# A cross-sectional mixed methods study protocol to generate learning from patient safety incidents reported from general practice

**DOI:** 10.1136/bmjopen-2015-009079

**Published:** 2015-12-01

**Authors:** Andrew Carson-Stevens, Peter Hibbert, Anthony Avery, Amy Butlin, Ben Carter, Alison Cooper, Huw Prosser Evans, Russell Gibson, Donna Luff, Meredith Makeham, Paul McEnhill, Sukhmeet S Panesar, Gareth Parry, Philippa Rees, Emma Shiels, Aziz Sheikh, Hope Olivia Ward, Huw Williams, Fiona Wood, Liam Donaldson, Adrian Edwards

**Affiliations:** 1 Primary Care Patient Safety (PISA) Research Group, Primary and Emergency Care Research (PRIME) Centre Wales, School of Medicine, Cardiff University, Wales, UK; 2 Department of Family Practice, Faculty of Medicine, University of British Columbia, Vancouver, Canada; 3 Australian Institute of Health Innovation, Macquarie University, Sydney, New South Wales, Australia; 4 Centre for Medical Informatics, Usher Institute of Population Health Sciences and Informatics, The University of Edinburgh, Scotland, UK; 5 Australian Patient Safety Foundation, Adelaide, Australia; 6 Division of General Practice, College of Community Health Sciences, University of Nottingham, Nottingham, UK; 7 Department of Anesthesia, Boston Children's Hospital, Boston, Massachusetts, USA; 8 Harvard Medical School, Boston, Massachusetts, USA; 9 Institute for Healthcare Improvement, Cambridge, Massachusetts, USA; 10 Division of General Internal Medicine, Brigham and Women's Hospital, Boston, Massachusetts, USA; 11 Department of Surgery and Cancer, Imperial College London, London, UK

## Abstract

**Introduction:**

Incident reports contain descriptions of errors and harms that occurred during clinical care delivery. Few observational studies have characterised incidents from general practice, and none of these have been from the England and Wales National Reporting and Learning System. This study aims to describe incidents reported from a general practice care setting.

**Methods and analysis:**

A general practice patient safety incident classification will be developed to characterise patient safety incidents. A weighted-random sample of 12 500 incidents describing no harm, low harm and moderate harm of patients, and all incidents describing severe harm and death of patients will be classified. Insights from exploratory descriptive statistics and thematic analysis will be combined to identify priority areas for future interventions.

**Ethics and dissemination:**

The need for ethical approval was waivered by the Aneurin Bevan University Health Board research risk review committee given the anonymised nature of data (ABHB R&D Ref number: SA/410/13). The authors will submit the results of the study to relevant journals and undertake national and international oral presentations to researchers, clinicians and policymakers.

**Strengths and limitations of this study**

- Incident reporting systems can be limited by the quality of data, particularly under-reporting, selective-reporting, incomplete-reporting and incident non-detection.
- Insights from exploratory descriptive statistics and thematic analysis will be combined to identify priority areas for future intervention.
- Our findings will be inductive and hypothesis generating.

## Introduction

Primary care poses unique challenges for the design of better quality systems of care delivery.^1–3^ To date, the focus on patient safety research has largely been within hospital settings and given the different case-mix considerations and the approach to care provision between hospitals and general practice the ability to transfer lessons to primary care has been limited.^1^ Despite 90% of healthcare interactions with healthcare professionals occurring in primary care settings in the UK, little is known about the possible risks to patients and their impact on patient health.^1^ ^3^ Systematic reviews of the primary care patient safety literature highlight a paucity of empirical work that explores the relationship between cause (error) and effect (harm), and the underlying system failures.^1^ ^4^

Established methods for examining healthcare safety, for example case note review, root cause analysis or incident reporting, provide different and incomplete observations of the underlying problems. Incident reporting systems have previously been used to identify priority areas and generate recommendations to improve care quality and safety at a local and national level.^5–8^ In 2003, a major investment was made in the National Reporting and Learning System (NRLS) to better understand incidents occurring in England and Wales. Each hospital and healthcare facility has a reporting system that collects paper or e-incident forms. Since 2004, NHS organisations in England and Wales have uploaded their incidents to the NRLS central database. Around 100 000 incidents a month are uploaded, making it the most comprehensive system in the world. The NRLS has informed multiple learning outputs including Rapid Response Reports, Patient Safety Alerts, and Safer Practice Notices.^9^ Despite these initiatives, incident reporting systems have gained little respect from the health information and research communities.^7^ ^10^ Incident reporting is underutilised in general practice which currently contributes to less than 1% of reports.^9^

The NRLS contains information about incidents with ‘free-text’ descriptions of the events, perceived contributing factors, and plans to minimise risk of reoccurrence. Over 40 000 reports from general practice in England and Wales have been submitted to the NRLS in the past decade, and these have never previously been systematically analysed. Such incidents permit a retrospective ‘window’ on the healthcare system, providing an opportunity for directing improvement initiatives by identifying weaknesses in the system that are leading to errors and harms occurring during clinical care delivery.^11^ Large-scale incident analysis is an under-exploited area within primary care patient safety, and should serve to demonstrate the value of safety monitoring, and emphasise the benefits of an effective reporting system for healthcare professionals, managers, leaders and patients in the NHS.

## Aims and objectives

We will undertake a mixed-methods study to characterise the nature and range of incidents reported from general practice in England and Wales, in order to:

1. Develop a classification using empirical evidence from reports.
2. Describe the relative frequency of different types of incidents.
3. Describe incident characteristics such as patient age, geography and level of patient harm.
4. Determine which characteristics are associated with different degrees of patient harm.
5. Map relationships between themes, as well as categories of incidents and potential contributory factors, and elicit possible areas with opportunity for intervention.

## Methods and analysis

### Data source

The definition of a patient safety incident in the NRLS is “any unintended or unexpected incident that resulted in or could have resulted in harm to one or more patients receiving state funded care”.^9^ Reporting incidents that resulted in severe harm or death of a patient became mandatory in June 2010; however, before this all reporting was voluntary, and remains so for incidents resulting in no, low or moderate harm.

Healthcare professionals have a duty to report incidents to healthcare organisations’ incident management systems. These are anonymised and uploaded to the NRLS. Each report contains categorical information about location, patient demographics and reporter perception of severity of harm–collected in a structured report form–as well as free-text descriptions of the incident, potential contributory factors and planned actions to prevent re-occurrence. The free text description, where the reporter is asked to describe what happened and why they think it happened, offers a rich body of qualitative data for identification of areas for improvement. These descriptions offer insight into the harms occurring or detected by healthcare professionals working in general practice from their perspective.

For more detail about the NRLS, Donaldson *et al*^12^ have described it in detail, including its current management in England and Wales.

### Study design

We will undertake a cross-sectional mixed methods study of reports that includes a thematic analysis informed by an exploratory data analysis.^13^ ^14^

#### Study setting

Incident reports will be received from 571 different locations such as Health Boards (formerly Local Health Boards) in Wales and Clinical Commissioning Groups (formerly Primary Care Trusts) in England.

#### Sample selection

Incidents received by NRLS between April 2005 and September 2013 from general practice will be considered as the complete data set (n=42 729 reports).

Given the inductive and exploratory nature of the study, we will analyse all incidents resulting in severe harm or death in the data set and a random sample of 12 500 non-fatal reports. To ensure results in our sample are current, a weighting will be applied to the random sample so that recent reports (2012-onward) are more prioritised than reports from previous years (2005–2009; 2010–2011), as well as increasing proportions from no harm, low harm and moderate harm, respectively. Following removal of all reports with a level of harm of severe and death, approximately 15%, 30% and 60% of each stratum will be drawn using a simple random sample without replacement. The probability of drawing a report is twice as likely in group 2 compared to group 1 (least recent and increasing proportions by level of harm from no harm to moderate harm), and four times more likely in group 3 (most recent and increasing proportions by level of harm from no harm to moderate harm) compared to group 1; this results in a data set with 12 500 reports see table 1.

**Table 1:** Study sample described by report period and level of harm

Reporting period	Group	Group size, N	Level of Harm			Total
			None	Low	Moderate	
April 2005–2009	1	18 039	2162	846	631	*3639*
2010–2011	2	12 660	2237	894	770	*3901*
2012–September 2013	3	11 198	2292	1721	947	*4960*
			*6691*	*3461*	*2348*	12 500

#### Classification system and reviewer training

**Figure 1:**
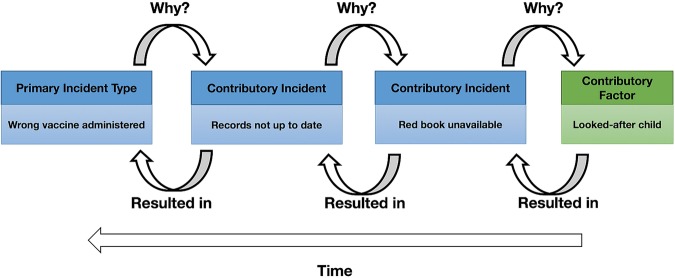
Example of codes from the Primary Care Patient Safety (PISA) Classification System using the Recursive Model for Incident Analysis.

The analysis of safety incident reports has largely been organised and managed by safety classification systems.^15–31^ Several patient safety classifications were reviewed and considered for inclusion,^4^ ^32–35^ including those developed for general practice.^34^ ^36–40^ These classification systems provided considerable guidance for shaping the scope of the system needed. To chronologically model the sequence of events culminating and contributing to an incident as per the classification rules of the Recursive Model of Incident Analysis (see figure 1 and see online supplementary appendix 1),^41^ a more granular framework is required. Therefore, we will empirically develop our own classification system to undertake a detailed description of incidents, including those that are complex in nature involving a sequence of events contributing to and culminating in the incident. The classification system will incorporate multiple coding frameworks. Based on the WHO International Classification for Patient Safety (WHO ICPS), four independent classes to describe the incident, its contributory factors and type of, and level of harm, will be developed using an iterative approach to create the Primary Care Patient Safety (PISA) Classification System.^42^

**Figure 2:**
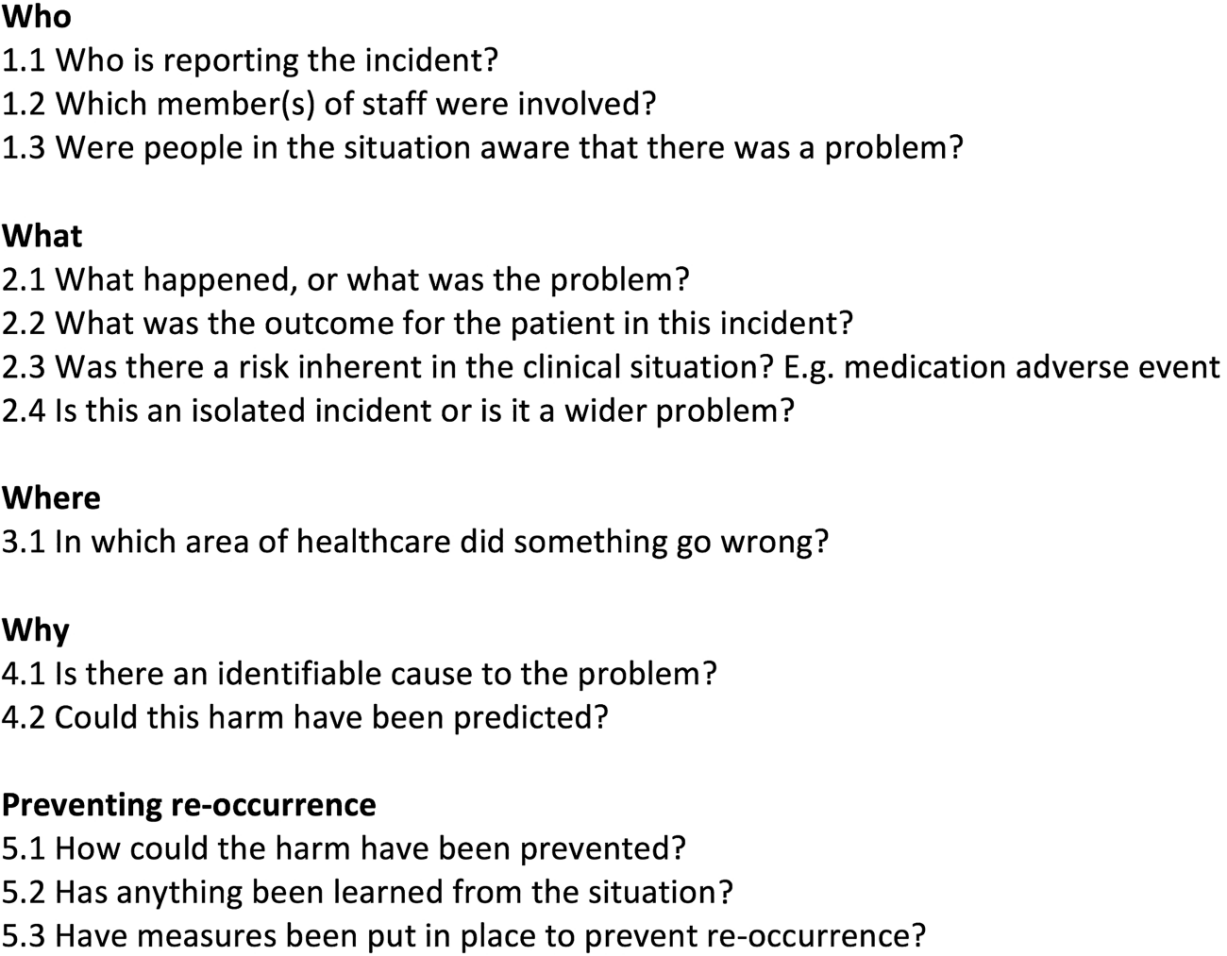
Checklist of questions for coding orientation.

A multidisciplinary team of clinicians will be recruited as incident reviewers. They will receive training in incident analysis, classification, root cause analysis and human factors in healthcare and undergo simulation with a practice data set. During the training period, to focus reviewers on the relevant content of interest, they will be required to identify in each incident the criteria outlined in figure 2. These criteria were developed by content analysis of 500 randomly sampled incidents by ACS and HOW. Early stages of familiarisation with the data by reading the reports and use of a priori codes from pilot work, will guide the iteration of the frameworks (discussed in more detail later).^43^ The reviewers’ interpretations will be informed by tacit knowledge, clinical expertise and the human factors training received to guide ‘sensemaking’, defined as “the active process of assigning meaning to ambiguous data”, in order to identify the learning that can be used to inform improvements in clinical care.^44^ ^45^ Once >70% agreement (κ statistic) between reviewers and an experienced coder (HW) is achieved, the reviewers will be able to code the study data.

#### Coding management system

To ensure our process is replicable for healthcare organisations to consider adopting our classification system, we have decided not to use an existing qualitative data analysis management software tool. In addition, given the distributed and international nature of the project (UK, USA and Australia), we have developed a bespoke solution to support the iteration of frameworks and provide secure access to numerous concurrent reviewers regardless of geographical location. This classification system will be made publicly available. The system is comprised of a backend database system and a web-based portal. The backend database has been built on Microsoft SQL Server 2014, with custom SQL algorithms to provide, for example, live concordance checks between reviewers’ double coding. The web front end was produced using a customised version of Portofino 4.1.1, an open source web framework written in Java.

#### Data analysis

There are three stages of work planned:

- *Stage 1*: *Familiarisation and data coding—*which involves reading the free-text of the report and applying codes to describe incident type, potential contributory factors, level and type of harm.
- *Stage 2*: *Generation of data summaries—*using descriptive statistical analysis.
- *Stage 3*: *Interpretation of themes and learning—*seeking to understand the most commonly identified patient safety themes, events leading up to it and reported contributory factors, and the contexts within which they occurred.

Each stage will now be considered in more detail.

Stage 1: Familiarisation and data coding

Reviewers will orientate themselves to the content by reading the incident report, which comprises several categorical variables and three categories of free-text data. Next, they will apply codes to each incident from the four classes—incident type, contributory factors, and type and degree of harm.

Coding large data sets requires effective teamwork in order to utilise the tacit knowledge and experience of multiple coders.^46^ To ensure validity and reliability of coding throughout the study, regular inter-coder reliability checks will be undertaken on a 20% random sample of each reviewer's coding quota for every 250 reports coded.^47^ κ statistics will be calculated for each principal incident type, defined as the incident that occurred just before the harm or potential harm. A κ of >0.7 is sought at checkpoints between reviewers for reports randomly identified for double-coding purposes; this is consistent with previous studies of a similar nature.^4^ Should the κ statistic fall below 0.7, both coders will meet (probably fortnightly) to discuss discrepancies and a re-test score will be calculated once both parties believe any issues of potential concern have been resolved. Where discrepancies cannot be resolved by discussion between reviewers, third person arbitration will be sought from a senior investigator (ACS/AE).^47^ It is envisaged all reviewers will code approximately equal shares of the total number of reports.

The codes within each class will be inductively amended throughout the process. Ideally, a codebook (a collection of coding classes) should be ‘all-inclusive’ with codes with definitions that are ‘mutually exclusive’.^48^ Where an existing code is not available to describe the incident characteristics, the study team will discuss at weekly coding meetings whether a new code is needed, or whether the definition of an existing code should be amended to be more inclusive. The study team is comprised of doctors, nurses, mixed methods researchers and patient safety experts. These meetings will also be used to discuss inter-coder agreement, and will attempt to resolve any issues that relate to understanding and application of specific codes.

Hypotheses that emerge from each step of analysis will be noted by reviewers during coding and analysis via electronic memos and discussed at weekly coding meetings. As codes are assigned, for example, ‘wrong dose administered’ and ‘wrong drug administered’, the codebook will be developed, and we anticipate the study team will begin to observe how cases cluster around particular codes or sets of related codes, and thematic groups will emerge, for example, ‘administration errors’, which will inform the development of each class.^49^ In addition, insights identified from review of descriptions of ‘planned actions to prevent future occurrences’, or any ‘protecting factors’ identified from the free-text description of the incident (eg, a parent or carer advocating on behalf of a patient and mitigating a more severe outcome), will be recorded in the memos and will also be used to inform discussions about recommendations for practice improvement.

Stage 2: Generation of descriptive summaries

We will describe and summarise the data, in order for it to inform subsequent hypothesis formation. The analysis will aim to describe the most commonly occurring incident types and codes, contributory factors and incident outcomes. Moreover, the analysis will explore high-level associations among these features. We will exclude reports that contain insufficient detail or do not describe a patient safety incident from these descriptive summaries.

The nature of our inquiry is inductive and guided by clinical expertise. Therefore, Exploratory Data Analysis (EDA) techniques will be applied, to produce for example, frequency tables, cross-tabulations, and bar charts, ready for interpretation and refinement through expert clinical guidance.^50^ As the purpose of our study is to generate learning to support healthcare professionals to improve the safety of care delivery, we recognise it is essential that the outcomes of the EDA are accessible and can provide a logical account of how we have identified the priority issues for possible intervention.

Frequency charts will enable us to identify the most common and most harmful reported incident types. Cross-tabulations between data variables (eg, age group, incident type, contributory factor, and incident outcomes), and between incident codes and contributory factor codes, will help to identify priorities (eg, vaccine errors in children) and clusters of common reported contributory events or factors for further inquiry by thematic analysis (Stage 3). We will explore whether the mandatory requirement for reporting incidents that result in severe harm or death since June 2010 has influenced reporting practices.

Stage 3: Interpretation of themes and learning

The purpose of our proposed thematic analysis^13^ is to deepen the analysis and interpretation gained in Stage 1 (description of characteristics of incidents) and Stage 2 (identifying patterns or recurring themes in the data) to identify and prioritise the most important patient safety problems, and characterise and interpret them to enable recommendation for improvements in practice (Stage 3).^13^

This is an EDA which collates relevant codes into themes and subthemes that describe the most common and most harmful reported safety incidents. Re-examination of these incidents in clusters of themes will provide opportunity to identify any contextual issues within each subset of data (eg, all reports describing moderate harms or worse following issues accessing clinical services for urgent assessment). The subsets of reports will be re-read by two clinicians (ACS and HW) and any iterations to the relevant themes or subthemes, as well as their definitions, will be discussed with the study team.

## Discussion

This will be the largest analysis of general practice patient safety incident reports undertaken. Leading experts recognise that despite limitations of reporting systems (underreporting, incomplete view of incident, and reporting biases) they provide multiple perspectives over time and form an integral part of routine monitoring in clinical practice.^11^ We will identify priority issues for improving the safety of healthcare delivery in general practice, and inform the development of a range of interventions and approaches to improve patient safety in this setting. By identifying priority issues and the key concepts for informing future improvement efforts, we anticipate our study will also provide momentum for promoting a reporting culture in general practice. Our findings will be hypothesis generating, inductive in nature, and require development and testing through future research and improvement efforts in clinical practice.

We will summarise our findings for dissemination to National Health Service organisations, and expect to make recommendations to enhance the future reporting and analysis of general practice incidents. Dissemination via presentations at national and international conferences and peer-reviewed journals is planned. We believe our findings could be relevant to patient advocacy organisations and special interest groups. We intend to organise training workshops with key stakeholders such as general practice registrars (those in their final year of training) and their trainers (those responsible for their supervision).

## Ethical considerations

Aneurin Bevan University Health board research risk review committee waived ethical approval (ABHB R and D Ref number: SA/410/13). Should we identify information within a report that raises professionalism or on-going patient safety issues, we will inform the relevant leads at the NHS commissioning board/NHS Wales so that they can appropriately deal with those concerns.
